# Author Correction: Brown adipose tissue-derived Nrg4 alleviates endothelial inflammation and atherosclerosis in male mice

**DOI:** 10.1038/s42255-022-00726-2

**Published:** 2022-12-26

**Authors:** Lingfeng Shi, Yixiang Li, Xiaoli Xu, Yangyang Cheng, Biying Meng, Jinling Xu, Lin Xiang, Jiajia Zhang, Kaiyue He, Jiayue Tong, Junxia Zhang, Lingwei Xiang, Guangda Xiang

**Affiliations:** 1 Department of Endocrinology, General Hospital of Central Theater Command, Wuhan, China; 2 The First School of Clinical Medicine, Southern Medical University, Guangzhou, China; 3 Endocrinology Department, The First Affiliated Hospital of the Army Medical University (Third Military Medical University), Chongqing, China; 4 Department of Hematology and Medical Oncology, School of Medicine, Emory University, Atlanta, GA USA; 5 Centers for Surgery and Public Health, Brigham and Women’s Hospital, Boston, MA USA

**Keywords:** Endocrine system and metabolic diseases, Cardiovascular diseases, Metabolism, Atherosclerosis

Correction to: *Nature Metabolism* 10.1038/s42255-022-00671-0, published online 18 November 2022.

In the version of this article initially published, two grant numbers in the Acknowledgements, now reading in part, “This work was supported by grants from the National Natural Science Foundation of China (nos. 81870573 and 81570730) to G.X.,” were incorrect, and have been amended in the HTML and PDF versions of the article.

